# Drip fertigation technique with planting models strategies trigger grain filling, endogenous hormonal changes and maize productivity under semi-arid regions

**DOI:** 10.3389/fpls.2026.1919758

**Published:** 2026-09-04

**Authors:** Jiaqi Wang, Jian Diao, Zhe Xu, Ning Ma, Shenzi Cheng, Ling Ma, Chunbo Teng

**Affiliations:** 1 Tianjin Lvyin Landscape And Ecology Construction Co., Ltd, Tianjin, China; 2 College of Life Sciences, Northeast Forestry University, Harbin, China; 3 College of Wildlife and Protected Area, Northeast Forestry University, Harbin, China; 4 College of Forestry, Northeast Forestry University, Harbin, China

**Keywords:** drip fertigation, endogenous hormonal, maize production, mulching, seed grain filling rate, water use efficiency

## Abstract

Mulch drip fertigation strategies have been widely adopted to improve maize productivity in semi-arid regions. However, the impact of the mulch-based drip fertigation on endogenous hormone dynamics in crops and grain yield has not been fully elucidated, and the biochemical mechanisms underlying yield improvement under different drip fertigation strategies remain unclear. Grain filling has a significant impact on maize production. The aim of this study was to explore the effect of plastic film mulching materials on maize grain filling under a drip fertigation strategy, and the relationship between this effect and changes in endogenous hormones. This study compared biodegradable film mulching (BM) with soil crust ridges (SR) under high (H), medium (M), and low (L) drip irrigation conditions, and measured that the grain filling characteristics and changes in grain endogenous hormones in various parts of maize ears during the growth stage. The results showed that the grain filling rate, dry matter plant^-1^, hormone changes, yield components, water use efficiency (WUE), and ET of maize under the BMH treatment were significantly higher than those under the SR treatment. BMH treatment significantly increased soil water storage significantly increased plant hormone contents such as abscisic acid (ABA), indole-3-Acetic acid (IAA), and zeatin + Zeatin Riboside (Z+ZR), and decreased the evolution rate of gibberellins (Gas) and ethylene, thereby increasing maize yield. These results indicate that BMH treatment improved the filling process, soil respiration rate, WUE, yield, and yield composition of maize. High drip fertigation with BM or SR treatment significantly affected endogenous hormone changes, thereby regulating the activity grain-filling period (AGP), maximum grain-filling rate (G_max_), maximum grain weight (W_max_), mean grain-filling rates (G_mean_) and occurrence time of maximal filling rate (T_max_) of maize. In addition, high drip fertigation and the BM strategy significantly increased soil water storage and soil respiration rate during the grain filling period. Based on these results, we conclude that BMH significantly improved soil water storage, thereby regulating the maize grain filling rate, which was significantly correlated with the balance of endogenous hormones in the grains.

## 1 Introduction

Rainfall is the primary water source for maize productivity in semi-arid regions (Ren et al., 2010; Zhang et al., 2021). However, insufficient and unstable precipitation often results in drought, leading low yields and occasionally complete crop failures (Wang et al., 2015; Li et al., 2020). To address the problem of insufficient water resources in these areas and increase maize yield, it has been essential to develop water-saving farming techniques that maximize the use of low rainfall and improve soil water storage (Zhang et al., 2011; Chen et al., 2023). Earlier research has shown that ridge mulching materials such as biodegradable films and soil crust ridges, can conserve rainwater, reduce evapotranspiration (ET), and improve soil respiration, thereby improving water use efficiency in maize (Peng and Horn, 2008; Martinez et al., 2011). Similarly, compared with soil ridges, biodegradable mulch significantly enhanced the average and maximum grain filling rates of maize (Ren et al., 2016; Fu et al., 2021). Many studies have revealed that drip fertigation strategies using mulching materials can reduce soil moisture evaporation (Li et al., 2001; Rusu et al., 2009). Furthermore, biodegradable mulch provides additional benefits, such as improving soil respiration and soil water storage (SWS) (Saneoka et al., 2004; Xie et al., 2008). Due to these advantages, biodegradable mulching materials have been widely used in semi-arid areas.

Several research studies indicate that ridge-covering materials have been widely developed and applied to dryland agricultural systems, greatly improving precipitation utilization and WUE (Guo et al., 2014; Ding et al., 2021). Furthermore, these mulching materials can retain sufficient rainwater during the main growth stages and reduce surface evaporation, thereby increasing WUE (Saini and Westgate, 2000; Nagaz et al., 2012). Earlier research has revealed that the impact of drip irrigation strategies on maize production may be associated with factors such as soil moisture content, and soil respiration rate ( Blanco-Moure et al., 2012). The yield potential of maize depends on grain weight, grains ear^-1^, and ear plant^-1^. Grain filling is the last stage of grain growth, after fertilization, the ovaries develop a caryopsis, ultimately determining grain weight (Zahedi and Jenner, 2003; Guo et al., 2022). However, in modern grain production systems, achieving optimal grain filling has become increasingly challenging (Xu et al., 2007; Zhao et al., 2014). Therefore, although it is important to determine whether and how mulch drip fertigation strategies affect maize grain filling, information regarding their effects on the grain filling process and the underlying biochemical mechanisms remains limited.

Water scarcity during the flowering and grain filling stages of maize can lead to a significant reduction in the maize production. These reductions are primarily due to oxidative damage, which reduces the seed setting rate and storage capacity of plants (Saini and Westgate, 2000; Wang et al., 2009). But, drought is often inevitable at the flowering and grain filling stages (Zahedi and Jenner, 2003; Liu et al., 2021). Mulch-based drip irrigation strategies provide an effective solution for improving water availability during these critical growth stages, thereby significantly improving soil respiration and maize production (Kang et al., 2002; Ran et al., 2023). Studies have revealed that soil respiration increases with increasing irrigation frequency, but excessive irrigation can suppress soil respiration (Yang et al., 2006; Shi et al., 2022). Geerts et al. (2008) reported that the levels of Z+ZR in developing maize grains showed a brief and significant increase after pollination, which coincided with the grain filling stage and maximum cell division stage in the endosperm. Higher concentrations of ABA and lower levels of ETH in maize kernels are associated with greater grain filling rates, and an increased in ABA to ETH ratio promotes grain filling (Ali and Thei, 2004; Farhad et al., 2024). In the early stage of grain filling, the content of IAA in high-quality grains is higher than that in low-quality grains (Wu et al., 2008; Gu et al., 2021). Furthermore, brassinosteroids and polyamines also regulate the grain filling process (Xu et al., 2007; Yang et al., 2008). These studies show that endogenous hormones have a significant effect on maize grain filling process; but, the association between changes in maize grain hormones levels and grain filling induced by mulch-based drip fertigation strategies remains unclear.

Previous studies have revealed that there are significant variations in the grain filling characteristics of grains located at different positions on the ear. In the early stage of grain filling, the content of Z+ZR and IAA are higher in superior grains (Wang et al., 2007; Guo et al., 2021). Research has found that higher ABA concentrations and lower ETH levels in superior maize grains are associated with their higher grain filling rates compared with inferior grains (Xin and Wang, 1998). Maize grains are highly sensitive to water stress (Nagaz et al., 2012). But, the variations in hormone levels among maize grains located at different positions in the ear, and their relationship with grain filling under mulch-based drip fertigation strategies, remain poorly understood. Previous studies have shown that the effects of mulching materials combined with drip fertigation strategies on maize production are closely associated with soil moisture (Zhao et al., 2014). In this study, different mulching materials combined with drip fertigation strategies were used to evaluate changes in endogenous hormone levels during the grain filling period. The purpose of this research was to explore the relationship between mulching materials and drip fertigation strategies on maize grain filling rate, soil water storage and to determine how changes in endogenous hormone levels regulate the grain filling process under different drip fertigation strategies.

## 2 Materials and methods

### 2.1 Site detail

**Figure 1:**
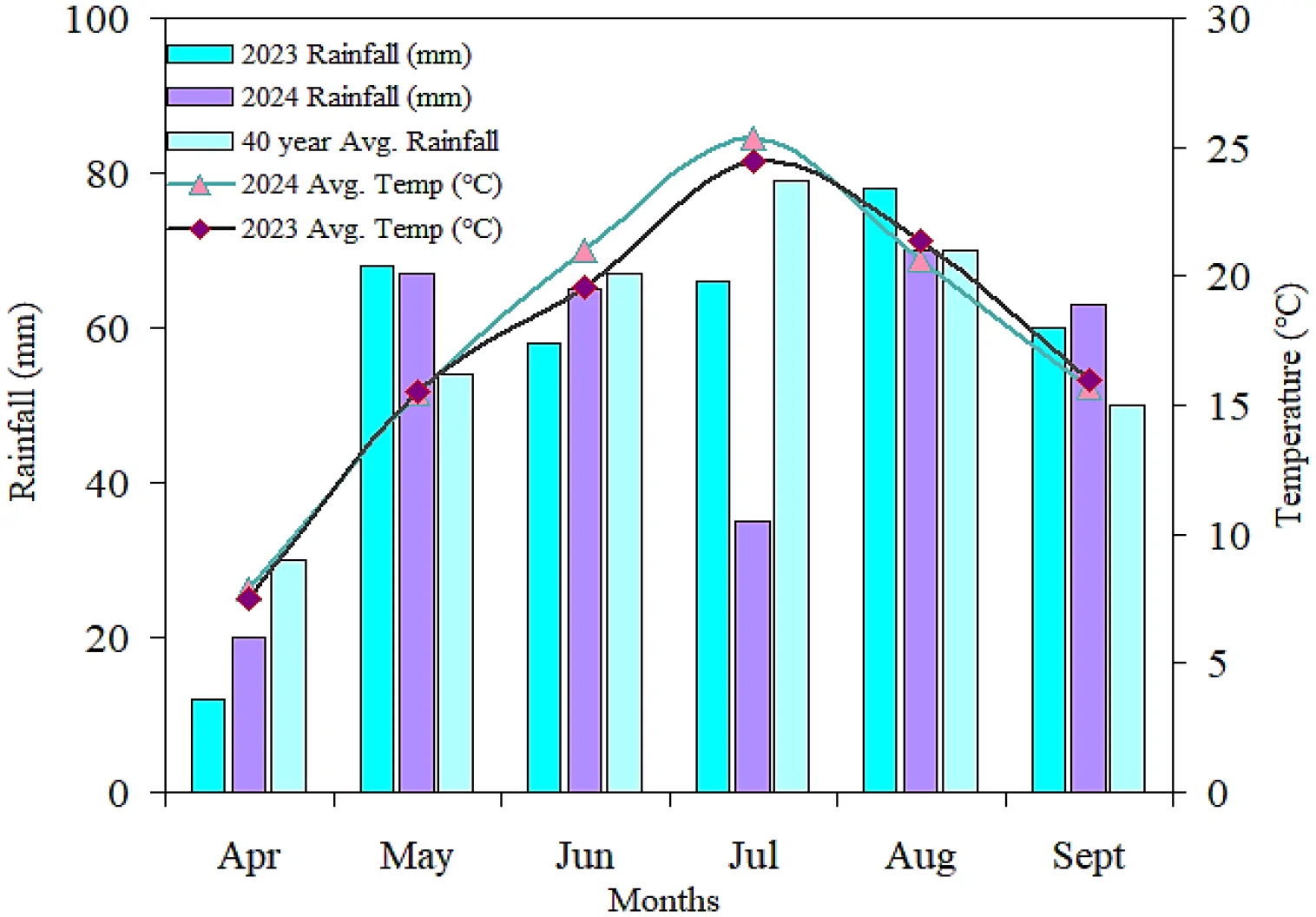
Monthly rainfall distribution during the maize-growing seasons in 2023 and 2024 at the experimental site.

This study was conducted during 2023 and 2024 in the Chahar Right Front Banner (Chayouqian Banner), Ulanqab City, Inner Mongolia Autonomous Region, P.R. China, located at 40.848711° N, 113.124087° E, with a semi-arid region. The annual average temperature is 6.3°C, the total sunshine hours are 3000 h yr-1, and the average rainfall are 450 mm yr-1. The rainfall from May to September, 2023 was 360 millimeters, compared to 353 millimeters in 2024. The monthly precipitation and temperature distribution are shown in **Figure 1**. The soil chemical properties are shown in **Table 1**.

**Table 1:** The chemical properties of experimental site of the soil layers (0-20 cm).

Year	pH	SOM (g kg^-1^)	TN (g kg^-1^)	TP (g kg^-1^)	TK (g kg^-1^)	AP (mg kg^-1^)	AK (mg kg^-1^)
2023	8.03	14.12	1.06	1.09	17.29	21.01	161.93
2024	7.99	15.01	1.07	1.05	17.38	20.88	163.01

SOM, soil organic matter; TN, total nitrogen; TP, total phosphorus; TK, total potassium; AP, available phosphorus; AK, available potassium.

### 2.2 Experimental detail

Used in a randomized complete block design (RCBD) with three replications and each experimental area was (20 × 3 m) 60 m^2^. The treatments consisted of two different ridges covering mulch materials: BM: biodegradable film mulch; SR: soil crust ridges; while, three drip fertigation strategies, H: 370 mm high drip fertigation; M: 75% of H, moderate drip fertigation; L: 50% of H, low drip fertigation. All experimental plots were irrigated with 50mm after sowing to achieve uniform germination. Throughout the growing season, single water applications (64, 45, and 27mm) were applied to H, M, and L treatments, totaling 5 times. Each plot is equipped with high-precision water meters (LXS-25, Ningbo), pressure gauges, and control valves to ensure accurate discharge and stable pressure. The biodegradable film was mulched on the ridge surface with the edges buried under in 3-5 cm depth soil. The biodegradable mulch film composed mainly of polybutylene adipate terephthalate (PBAT) and polylactic acid (PLA) was used. The film had a thickness of 0.010 mm (10 μm), and was supplied by Kingfa Sci. & Tech. Co., Ltd., Guangzhou, China. The soil crust ridges (SR) was compacted manually with wooden blocks. The Dafeng-30 maize cultivar was grown on May 10, 2023, and May 11, 2024, with a row spacing of 60 cm and 75 thousand standard plant populations. Maize was harvested on September 20, 2023, and September 19, 2024. While, 160 and 40 kg ha^-1^ of N were applied through drip fertigation system. Nitrogen was applied in two split applications during the jointing and flowering stages. For each fertigation event, the required quantity of fertilizer was completely dissolved in clean water and injected into the irrigation pipeline using a fertilizer injector. Before fertilizer injection, the drip irrigation system was operated with clean water to establish uniform water flow throughout the field. After the fertilizer solution had been delivered, irrigation with clean water continued to flush the pipelines and facilitate fertilizer movement into the active root zone, thereby minimizing nutrient accumulation within the drip lines and reducing potential nitrogen losses.

#### 2.3.1 Soil water storage (SWS)

Soil water storage was calculated by using following **Equation 1**:

(1) SWS=C×ρ×H×10

where C is the gravimetric water content (%); ρ (g cm^-3^) is the bulk density; H (cm) is the soil depth with 20 cm interval.

The evapotranspiration (ET) rate was calculated by using following **Equation 2**:

(2) ET = (P+ I + F) – (D + R -ΔW)

where P (mm) is the total precipitation; I (mm) is the drip irrigation; F is the upward flow into the root zone; D is the downward drainage out of the root zone; R is the surface runoff from each plot, and ΔW (mm) is the soil moisture content for the 0–200 cm soil depths between planting time and maturity stage, or between the growth stages (3).

(3) WUE = Y/ET

Water use efficiency (WUE, kg ha^-1^ mm^-1^) was calculated using the above **Equation 3**. Where Y is the grain yield (kg ha^-1^), and ET is the total evapotranspiration (mm) over the growing season.

Soil respiration rates were recorded in the field using an LI-8100 Automated Soil CO_2_ Flux System connected with temperature and moisture sensors at the flowering stage, grain filling stage and maturity stage of the maize crop.

SOM was measured by the potassium dichromate oxidation method. TN was determined using the Kjeldahl digestion method. TP was measured following acid digestion using the molybdenum blue colorimetric method. TK was determined after acid digestion using flame photometry (or inductively coupled plasma optical emission spectrometry, ICP-OES, if applicable). AP was extracted with sodium bicarbonate (Olsen method) and quantified by the molybdenum blue colorimetric method. AK was extracted with 1.0 mol L^-^¹ ammonium acetate (pH 7.0) and determined using a flame photometer.

#### 2.3.2 Grain filling and hormones

One hundred ears that silked on the same day were selected and tagged in each plot. Three tagged ears from each plot were sampled at 3 days intervals from silking to maturity. The grains of the ear were divided into three parts, on average, according to the ear length, and the grain growth was removed from the various parts of the ear. The grains from each ear were divided into upper grains, middle grains and basal grains.

Using the Richards (1959) **Equation 4** for fitted the grain filling data:

(4) $$W=\frac{A}{{\left(1+Be^{-kt}\right)}^{\frac{1}{N}}}$$

The grain filling rate (G) was determined by using following **Equation 5**:

(5) $$G=\frac{AkBe^{-kt}}{N{\left(1+Be^{-kt}\right)}^{\left(\frac{N+1}{N}\right)}}$$

where, W, the grain weight (mg); A, the final grain weight (mg); t, time after anthesis (d); B, k and N, coefficients determined by regression. The active grain-filling period was defined as the period when W was between 5% (t1) and 95% (t2) of A. The average grain-filling rate during this period was therefore calculated from t1 to t2. W_max_: the maximum grain weight; G_mean_: mean grain-filling rates; G_max_: maximum grain-filling rates; T_max_: occurrence time of maximal filling rate; AGP: activity grain-filling period.

Yang et al. (2001) used a method of purification and extraction of ABA, Z+ZR, and GAs (GA1 + GA4) contents. Grind 0.5 grams of seed samples in a cold mortar containing 5 milliliters of 80% (v/v) methanol extraction medium, which contains 1 mole of L^-1^ butylhydroxytoluene. Incubate methanol extract at 4 °C for 4 hours, then at 4 °C, 10000 × Centrifuge under g for 15 minutes. Pass the supernatant through a Chromosep C18 column (C18 Sep-Park Cartridge, Waters Corp, USA) for enzyme-linked immunosorbent assay (ELISA) evaluation. The level of ethylene evolved from the analyzed grains was determined according to the method described by Beltrano et al. (1994), with modifications. The quantification method for Z + ZR, GAs (GA1 + GA4), IAA, and ABA by ELISA was described previously (Yang et al., 2001). The rate of ethylene evolution was expressed as a function of the unit fresh weight (FW).

#### 2.3.3 Dry matter and maize yield

Randomly select 6 plants from each plot with 3, 6, and 11 leaves, silking, grain filling and harvesting stage to determine the dry matter plant^-1^. Manually harvest two rows of maize from the center of each plot, including a combination of ridges and furrows. You can measure their 30 plants randomly by selecting crop and yield components such as ear diameter, ear length, grain ear^-1^, grain weight ear^-1^, and 1000 grain weight.

### 2.4 Statistical analysis

A randomized complete block design (RCBD) was used under the two factorial analysis of variance (ANOVA) (Planting models × Drip fertigation technique) by using the SPSS 13.0 program (SPSS Inc., Chicago, IL, USA). The three replicates data for each treatment were analyzed, and significant differences between treatments were determined based on the least significant difference (LSD) test. Analyze the data for each sampling event separately. Compare the average values of each treatment based on the least significant difference test (LSD 0.05). The data obtained from each sampling event (growth stage) were analyzed separately. The year data was analysis separately. Data analyses and all figures were made by using Excel 2010, and Sigma Plot 12.5. The ANOVA assumptions were met without any transformations.

## 3 Results

### 3.1 Soil water storage (SWS) and ET rate

In 2 successive years, the SWS profiles in the 0–200 cm soil layers were determined frequently under different treatments throughout the entire growth season of maize (**Table 2**). In the two study years, there were significantly different in SWS, significantly due to differences in rainfall and its distribution events, the soil moisture content under various mulching materials using drip irrigation strategies has changed (**Table 2**). The WUE of crops high with crop growth, but no drought conditions were recorded under BMH and BMM treatments. Under BMH treatment at a soil depth of 0-200 cm, the SWS of different growth stages (excluding SS stage) was significantly higher than that of all treatments. From SS phase to MS phase, there is a significant increase in SWS for each treatment. During the FS phase, the average data for two years showed a significant increase in SWS under BMH and BMM treatments compared to SRM and SRH treatments. Under different mulching droplets, irrigation and fertilization strategies have significant maximum SWS throughout the entire growth stage. From JS to FS stage, there were important variances in SWS between different treatments in the two study years. With the improvement of drip irrigation fertilization levels under different film-covering materials, SWS has increased throughout the entire growth stage of maize.

**Table 2:** Effects of different treatments on soil water storage of maize during 2023 and 2024 year.

Treatments	Soil water storage (mm)					
	SS	TLS	JS	FS	GFS	MS
2023						
BMH	175a	208a	234a	240a	244a	247a
BMM	174a	203a	227a	232b	236b	239b
BML	174a	189c	207c	217c	211d	207d
SRH	175a	196b	218b	227b	226c	221c
SRM	176a	189c	214b	220c	226c	209d
SRL	174a	182d	199d	203d	206d	200e
ANOVA						
CM	ns	*	*	*	*	*
DI	ns	*	*	*	*	*
CM * DI	ns	ns	ns	ns	ns	ns
2024						
BMH	171a	210a	239a	236a	243a	247a
BMM	169a	205a	222b	228b	235b	239b
BML	169a	191c	207c	213c	210d	214d
SRH	168a	198b	218b	224b	230b	226c
SRM	171a	191c	214c	216c	225c	209d
SRL	169a	184d	200d	203d	205d	200e
ANOVA						
CM	ns	*	*	*	**	*
DI	ns	**	*	*	*	*
CM * DI	ns	ns	ns	ns	ns	ns

BMH: biodegradable film mulching on ridges with high drip fertigation irrigation; BMM: biodegradable film mulching on ridges with 75% of H, moderate drip fertigation irrigation; BML: biodegradable film mulching on ridges with 50% of H, low drip fertigation irrigation; SRH: soil crust ridges with high drip fertigation irrigation; SRM: soil crust ridges with 75% of H, moderate drip fertigation irrigation; SRL: soil crust ridges with 50% of H, low drip fertigation irrigation. SS: sowing stage; TLS: three leaf stage; JS: jointing stage; FS: flowering stage; GFS: grain-filling stage; MS: maturity stage. Followed by different small letters within a column indicate significantly different at the 5% probability level (LSD; n=3). ** Significant differences at p< 0.01; * significant differences at p< 0.05; ns indicates non-significant difference.

During the maize growing season, there are significant variances in ET among different treatments. Under the ridge and furrow (RF) system, the use of biodegradable film-covered ridges and drip irrigation strategies resulted in the lowest ET rate compared to those under soil crust ridges. In 2023, ET significantly decreased with the increase of BMH (P<0.05); BMM; and BML; Compared with SRL treatment, SRH and SRM treatments reduced by 15.2%, 7.1%, 0.8%, 10.3%, and 9.4%, respectively. In 2024, ET significantly decreased with the increase of BMH (P<0.05); BMM; and BML; Compared with SRL treatment, SRH and SRM treatments reduced by 12.9%, 8.8%, 5.4%, 9.4%, and 3.8%. In the 2-year experiment, there was no significantly variance in ET between BML and SRL treatments.

### 3.2 Characteristics of grain-filling rate

Two years of field research have confirmed that under different film mulching materials, W_max_, G_mean_, and G_max_ increase with the increase of fertilization level under film mulching drip irrigation (**Table 3**). During 2023, there was no significantly variance in W_max_, G_mean_, and G_max_ between BMH and BMM treatments under different drip irrigation levels. However, under different film mulching materials, W_max_, G_mean_, and G_max_ under low drip irrigation levels were significantly lower than those under high and medium drip irrigation levels. In 2023, under higher drip irrigation levels using biodegradable film mulch, W_max_, G_mean_, and G_max_ was significantly greater; however, there was no significantly variance between high and medium drip irrigation levels under BM and SR treatments. In the two year study, the maximum filling occurrence time (T_max_) under BMH and BMM treatments was much later than the rest of the treatments. However, in these two study years, there was no significantly change in AGP under BMM and SRM treatment. AGP increases with the improvement of drip irrigation levels under different film-covering materials. Under different mulching materials, the AGP under high drip irrigation fertilization level was significantly higher than that under low drip irrigation fertilization level.

**Table 3:** Effects of different mulch drip fertigation management strategies on grain-filling characteristics of maize in 2023 and 2024.

Treatments	W_max_ (mg)	G_mean_ (mg grain^-1^ d^-1^)	G_max_ (mg grain^-1^ d^-1^)	T_max_ (d)	AGP (d)
2023					
BMH	281.1a	7.0a	11.6a	27.4a	44.3a
BMM	278.6b	6.9b	11.4a	26.2a	40.0b
BML	268.8d	6.1c	10.8b	25.9b	38.4c
SRH	277.3b	7.0a	11.7a	25.8b	41.8b
SRM	275.6c	6.9b	11.0a	25.1b	38.6c
SRL	266.7d	6.0c	10.5b	24.7c	37.2d
ANOVA					
CM	*	*	*	*	*
DI	*	*	*	*	*
CM * DI	ns	ns	ns	ns	ns
2024					
BMH	276.6a	6.8a	10.7a	27.2a	42.3a
BMM	272.4b	6.6a	10.8a	26.2a	39.6c
BML	264.3c	6.1b	9.9b	25.4b	37.6d
SRH	271.2b	6.7a	10.7a	26.9a	40.5b
SRM	263.3c	6.1b	9.9b	25.4b	38.6c
SRL	261.7d	6.0b	9.3b	24.3c	36.4e
ANOVA					
CM	*	*	*	*	**
DI	*	*	*	*	*
CM * DI	ns	ns	ns	ns	ns

W_max_: the final maximum grain weight; G_max_: maximum grain-filling rates; G_mean_: mean grain-filling rates; T_max_: occurrence time of maximal filling rate; AGP: activity grain-filling period. Followed by different small letters within a column indicate significantly different at the 5% probability level (LSD; n=3). ** Significant differences at p< 0.01; * significant differences at p< 0.05; ns indicates non-significant difference.

**Figure 2:**
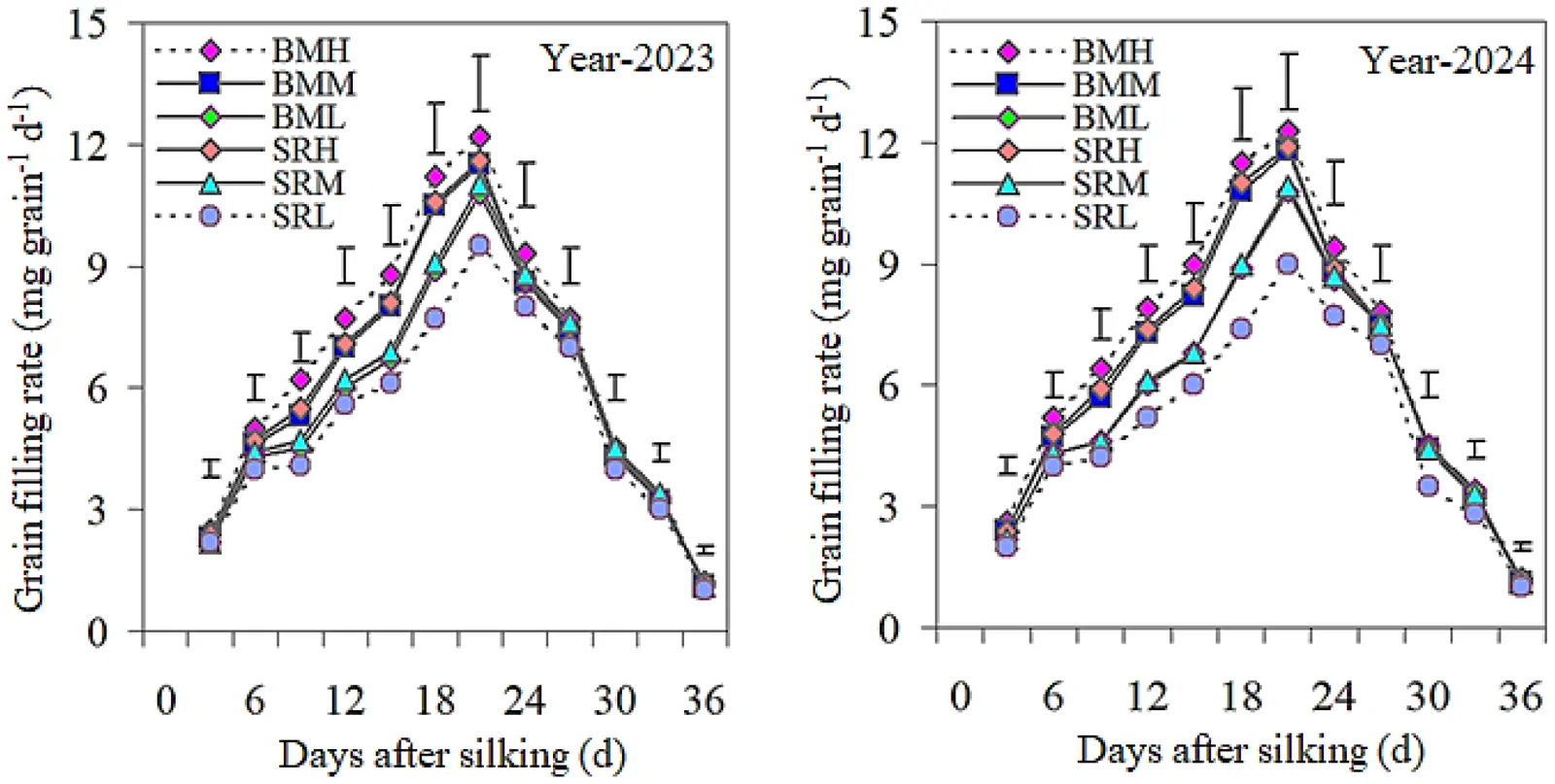
Effects of mulch drip fertigation strategies on grain filling rate under various mulching materials. BMH, biodegradable film mulching on ridges with high drip fertigation irrigation; BMM, biodegradable film mulching on ridges with 75% of H, moderate drip fertigation irrigation; BML, biodegradable film mulching on ridges with 50% of H, low drip fertigation irrigation; SRH, soil crust ridges with high drip fertigation irrigation; SRM, soil crust ridges with 75% of H, moderate drip fertigation irrigation; SRL, soil crust ridges with 50% of H, low drip fertigation irrigation. The vertical bars represent the LSD (n = 3).

The fertilization level of film mulching drip irrigation significantly affects the seed filling rate of maize. With the improvement of drip irrigation fertilization level, the grain filling rate showed an upward trend under various film covering materials (**Figure 2**). Compared with SR coverage, BM also significantly affects the seed filling rate. The seed filling process significantly enhanced in the early stage of silking and then reduced. Under BMH and BMM treatments, the grain filling rate was significantly maximum. The seed filling process of BM and SR treatments with high and medium drip irrigation fertilization levels was significantly greater as compared with SRL treatment 3 to 21 days after silking. The BM treatment with high or moderate drip irrigation fertilization levels showed the highest grain filling rate 21 days after silking.

### 3.3 Soil respiration rate

During the three main growth stages of two years, biodegradable film coverage on ridges with higher drip irrigation levels had a significantly impact on soil respiration rate (**Table 4**). There was no significantly variance in soil respiration rate between BMH and BMM during the FS, GFS, and MS stages. The average of two-year data shows that during the FS, GFS, and MS stages, the soil respiration of BMH was 22.8%, 37.2%, and 52.1% higher than that of BML treatment, respectively. Compared with SRL treatment, SRH treatment increased soil respiration by 42.1%, 41.6%, and 55.6% in the FS, GFS, and MS stages. There was no significantly variance in soil respiration between BM planting modes with high and medium drip irrigation levels at various stages of maize growth in the two study years.

**Table 4:** Effects of different treatments on soil respiration rate of maize during 2023 and 2024.

Treatments	Soil respiration rate (µmol m^-2^ s^-1^)					
	2023-Year			2024-Year		
	FS	GFS	MS	FS	GFS	MS
BMH	4.84a	3.80a	3.26a	5.19a	4.80a	4.71a
BMM	4.07a	2.68b	3.17a	5.07a	3.45b	2.81c
BML	3.34b	2.08b	1.71c	4.40b	3.32b	2.11c
SRH	4.56a	3.00a	2.98b	5.03a	4.66a	3.89b
SRM	3.77b	2.29b	2.06b	4.88b	3.31b	2.44c
SRL	2.42c	1.83c	1.24d	3.13c	2.64c	1.81d
ANOVA						
CM	*	*	*	*	*	*
DI	*	*	*	*	*	*
CM * DI	ns	ns	ns	ns	ns	ns

FS, flowering stage; GFS, grain-filling stage; MS, maturity stage. Followed by different small letters within a column indicate significantly different at the 5% probability level (LSD; n=3). ** Significant differences at p< 0.01; * significant differences at p< 0.05; ns indicates non-significant difference.

### 3.4 Hormonal changes

#### 3.4.1 Z + ZR and IAA contents

**Figure 3:**
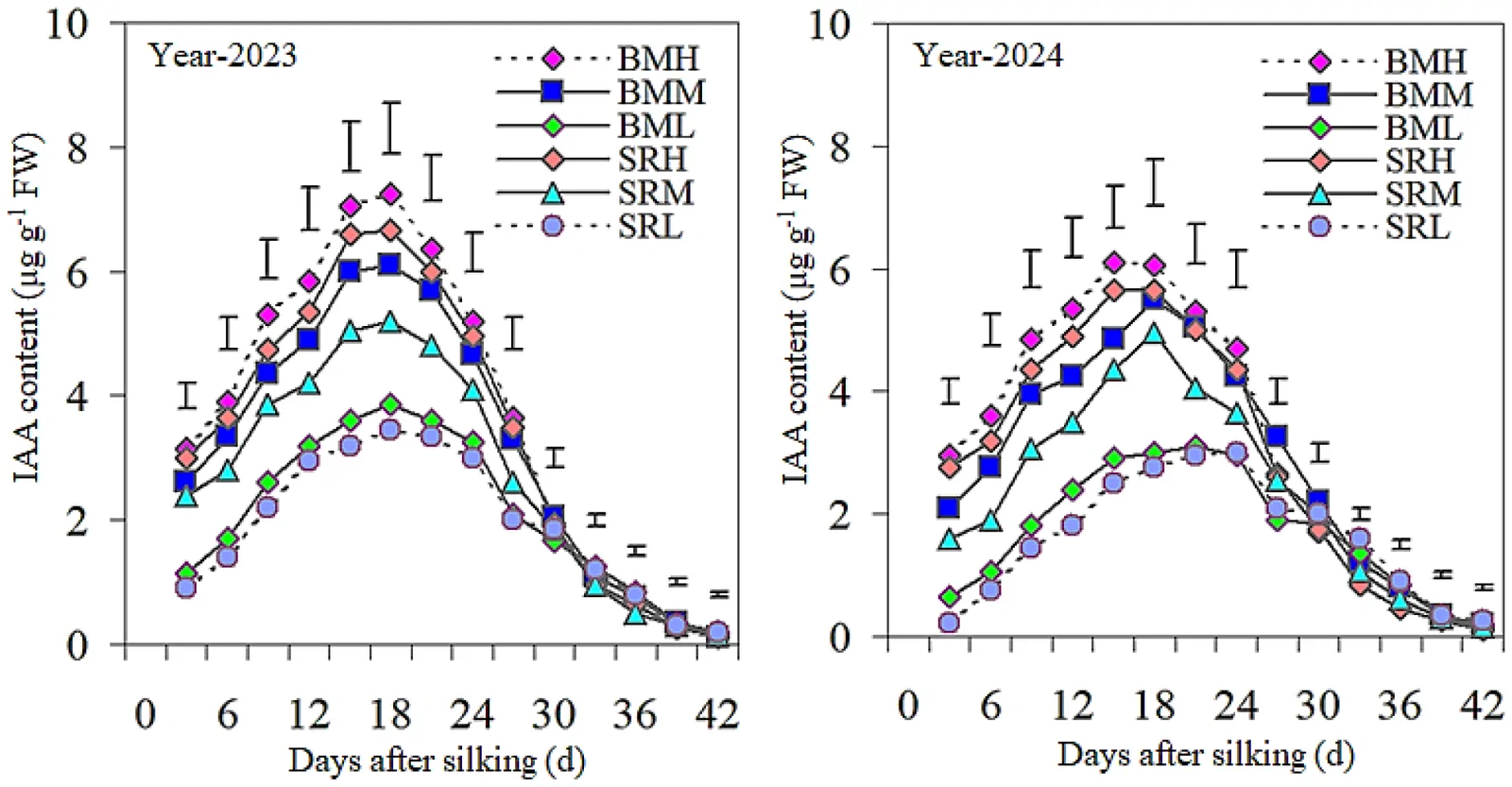
Effects of mulch drip fertigation strategies on IAA content in maize grains under various mulching materials. BMH, biodegradable film mulching on ridges with high drip fertigation irrigation; BMM, biodegradable film mulching on ridges with 75% of H, moderate drip fertigation irrigation; BML, biodegradable film mulching on ridges with 50% of H, low drip fertigation irrigation; SRH,: soil crust ridges with high drip fertigation irrigation; SRM, soil crust ridges with 75% of H, moderate drip fertigation irrigation; SRL, soil crust ridges with 50% of H, low drip fertigation irrigation. The vertical bars represent the LSD (n = 3).

**Figure 4:**
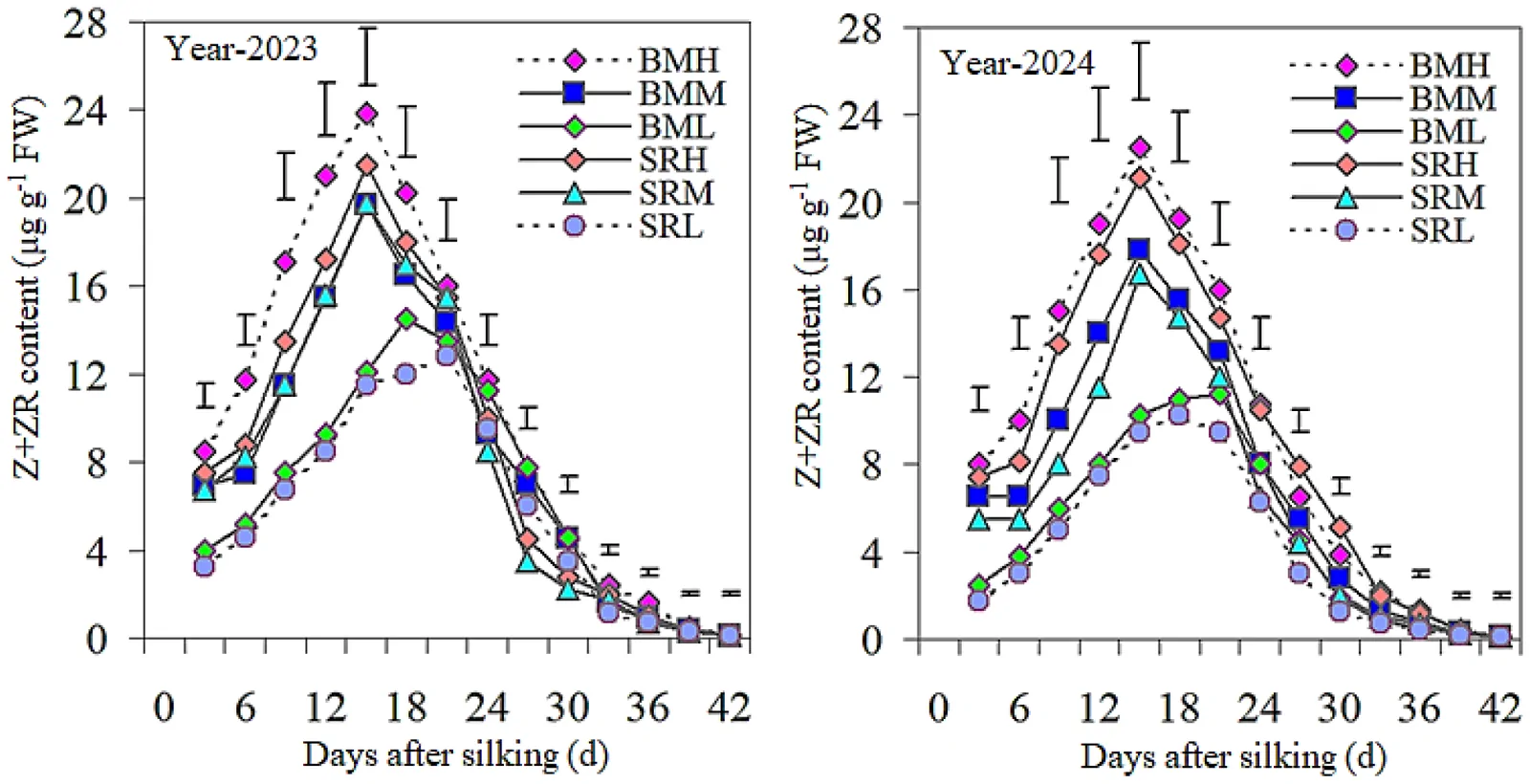
Effects of mulch drip fertigation strategies on Z+ZR content in maize grains under various mulching materials. BMH, biodegradable film mulching on ridges with high drip fertigation irrigation; BMM, biodegradable film mulching on ridges with 75% of H, moderate drip fertigation irrigation; BML, biodegradable film mulching on ridges with 50% of H, low drip fertigation irrigation; SRH, soil crust ridges with high drip fertigation irrigation; SRM, soil crust ridges with 75% of H, moderate drip fertigation irrigation; SRL, soil crust ridges with 50% of H, low drip fertigation irrigation. The vertical bars represent the LSD (n = 3).

The Z+ZR and IAA concentrations of maize vary similarly during the seed filling process. The Z+ZR and IAA content of maize increases briefly at the filling stage of the maize, reaches its maximum value 18 days after silking, and then decreases (**Figures 3**, **4**). In the early stages of grain filling, Z+ZR and IAA in seeds grow rapidly and then decrease. In BMH, Z+ZR and IAA improved significantly compared to other treatments. Different film-coated materials have a significantly effect on Z+ZR and IAA concentrations in different film-coated fertilizers. In BMH and SRH treatments, Z+ZR and IAA improved significantly compared to other treatments 3-18 days after silking. Furthermore, the peak values of Z+ZR and IAA in seeds treated with BMH were higher than in seeds treated with SRH and BMM. At 18DAS, the Z+ZR and IAA contents in the seeds of BM planting mode reached their maximum values, while at 21DAS, the Z+ZR and IAA in the seeds of BML and SRL reached their maximum values. The BM with a high drip irrigation level also significantly increased the Z+ZR and IAA in the early stage of grain filling. But, compared to SR planting technology, the peak values of Z+ZR and IAA under BM planting mode are synchronous.

#### 3.4.2 ABA content

**Figure 5:**
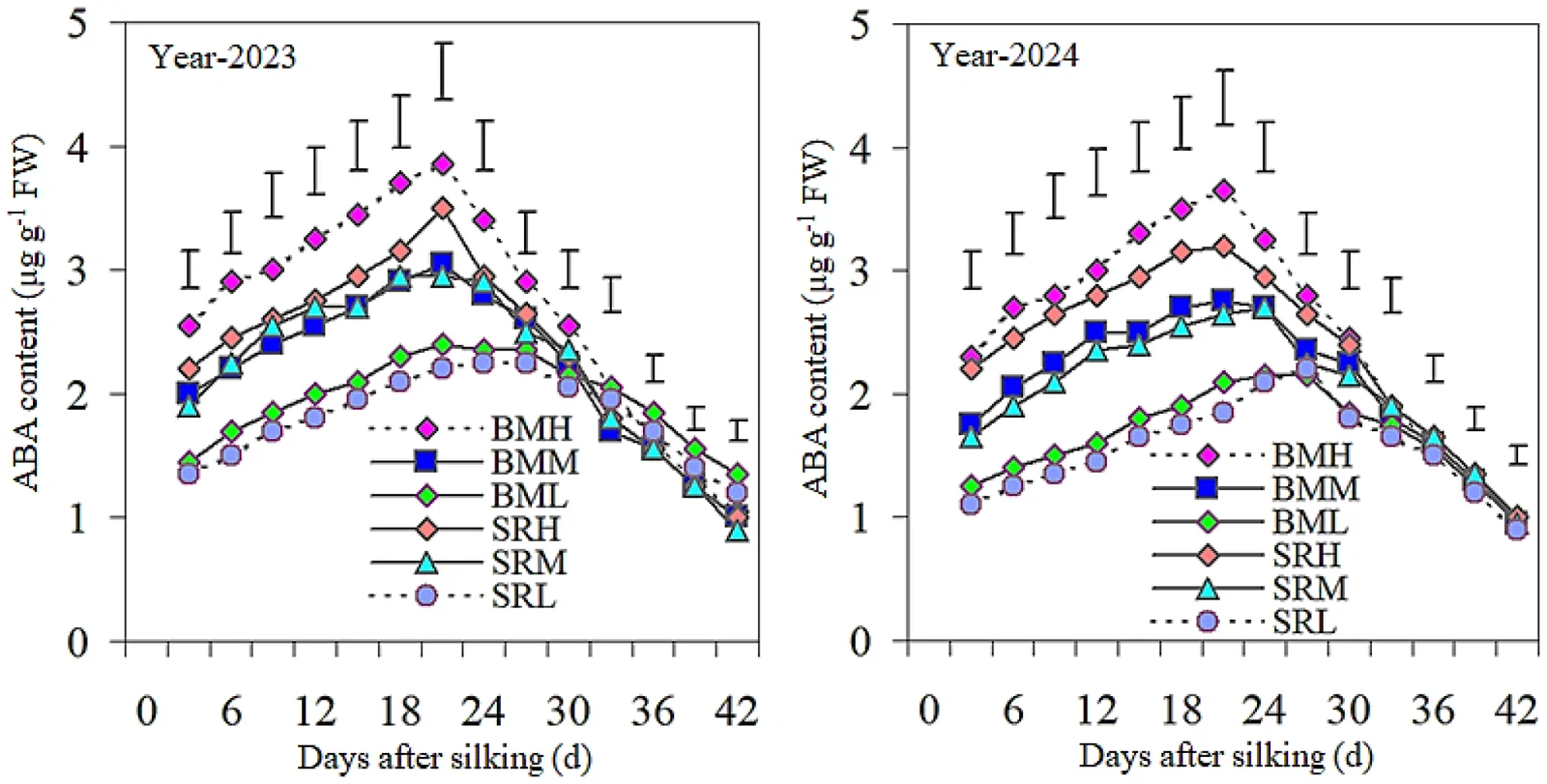
Effects of mulch drip fertigation strategies on ABA content in maize grains under various mulching materials. BMH, biodegradable film mulching on ridges with high drip fertigation irrigation; BMM, biodegradable film mulching on ridges with 75% of H, moderate drip fertigation irrigation; BML, biodegradable film mulching on ridges with 50% of H, low drip fertigation irrigation; SRH, soil crust ridges with high drip fertigation irrigation; SRM, soil crust ridges with 75% of H, moderate drip fertigation irrigation; SRL, soil crust ridges with 50% of H, low drip fertigation irrigation. The vertical bars represent the LSD (n = 3).

Maize seeds rapidly increase ABA in the early stage of grain filling, and then slowly decrease. As compared with SRL and BML treatments, the ABA content under BMH treatment was significantly higher (**Figure 5**). The ABA content significantly increased from 3 to 24 days after silking stage. Under the film drip fertilization irrigation strategy, different film covering materials have a significant impact on ABA content. Under high and medium drip irrigation fertilization levels, BM treatment significantly increased ABA content compared to SRL and BML treatments, respectively. However, under different film drop fertilization strategies, the effect of different film covering materials on ABA was not significant from 33 to 42 days after silking stage.

#### 3.4.3 GA and ETH evolution rate

**Figure 6:**
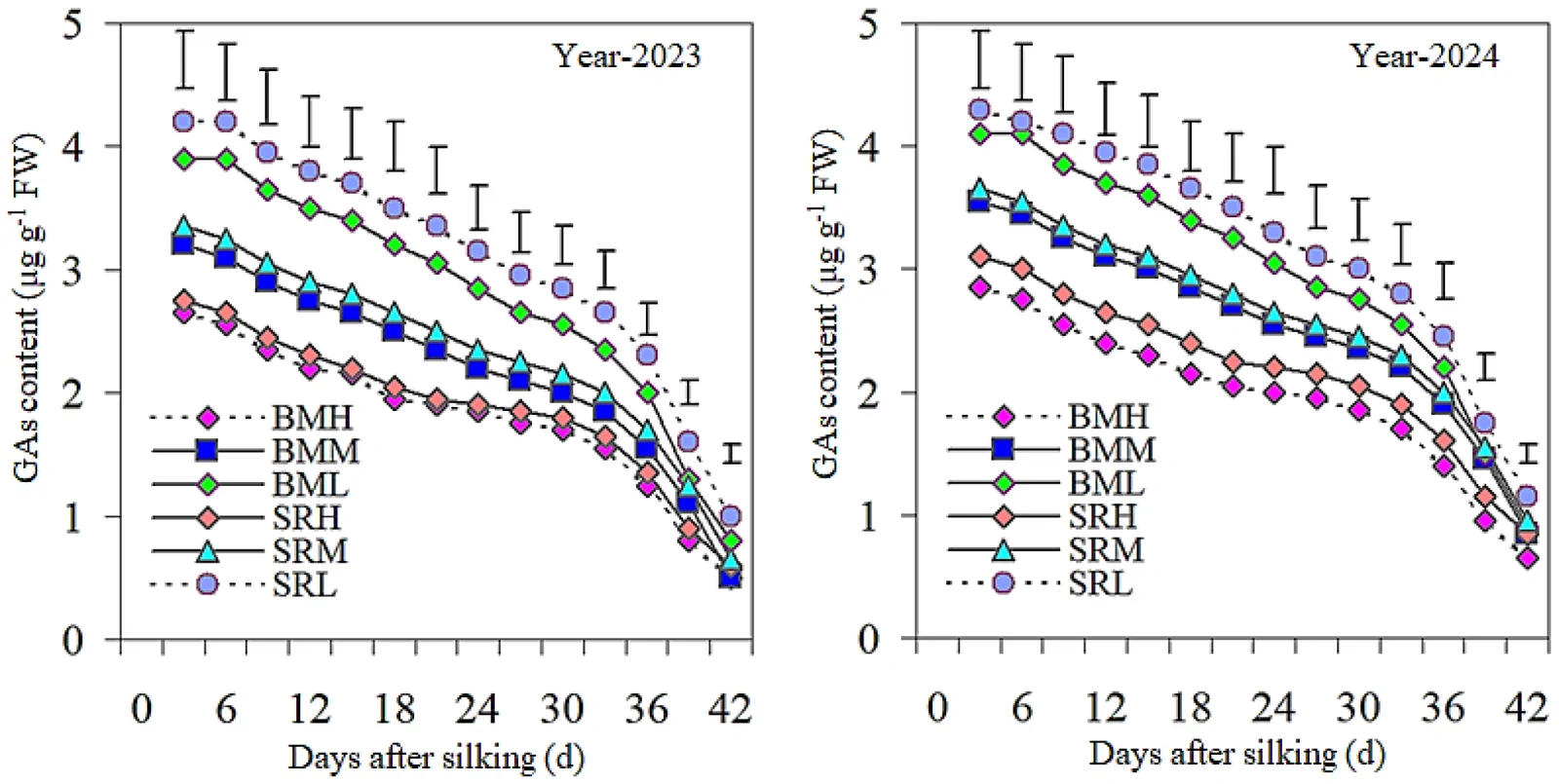
Effects of mulch drip fertigation strategies on GAs content in maize grains under various mulching materials. BMH, biodegradable film mulching on ridges with high drip fertigation irrigation; BMM, biodegradable film mulching on ridges with 75% of H, moderate drip fertigation irrigation; BML, biodegradable film mulching on ridges with 50% of H, low drip fertigation irrigation; SRH, soil crust ridges with high drip fertigation irrigation; SRM, soil crust ridges with 75% of H, moderate drip fertigation irrigation; SRL, soil crust ridges with 50% of H, low drip fertigation irrigation. The vertical bars represent the LSD (n = 3).

**Figure 7:**
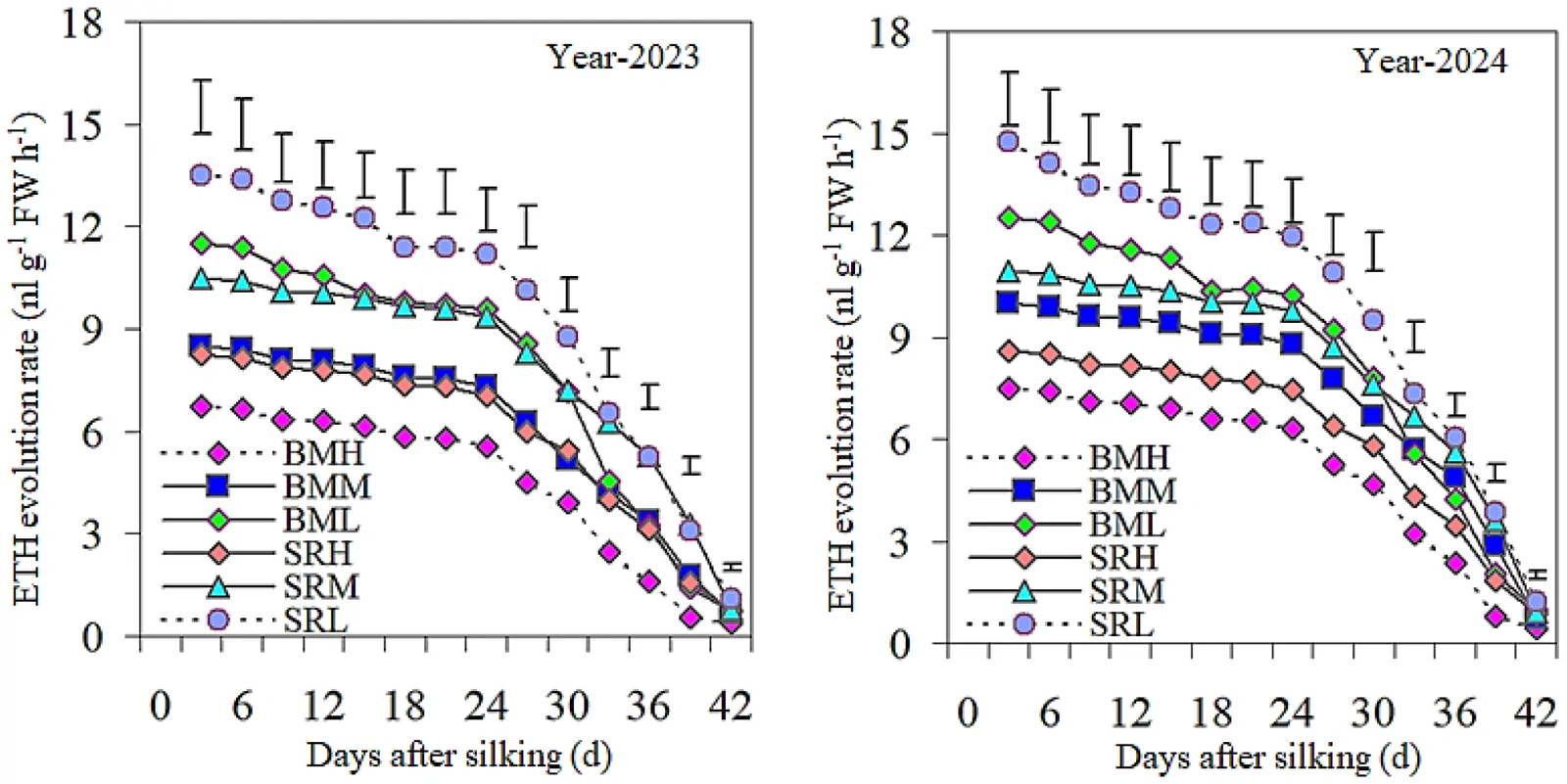
Effects of mulch drip fertigation strategies on ETH evolution rate in maize grains under various mulching materials. BMH, biodegradable film mulching on ridges with high drip fertigation irrigation; BMM, biodegradable film mulching on ridges with 75% of H, moderate drip fertigation irrigation; BML, biodegradable film mulching on ridges with 50% of H, low drip fertigation irrigation; SRH, soil crust ridges with high drip fertigation irrigation; SRM, soil crust ridges with 75% of H, moderate drip fertigation irrigation; SRL, soil crust ridges with 50% of H, low drip fertigation irrigation. The vertical bars represent the LSD (n = 3).

GA and ETH exhibit similar trends during the grain filling period. The GAs and ETH in grains gradually decrease throughout the filling stage (**Figures 6**, **7**). During the seed filling stage, the GAs and ETH of SRL and BML treatments were significantly greater than those of BMH and SRH. Compared with the BM treatment of low drip irrigation fertilization strategy, the SR treatment of low drip irrigation strategy significantly improved the evolution rate of GA and ETH. On the contrary, under high and medium drip irrigation fertilization levels, BM treatment significantly reduced GAs and ETH compared to SR treatment at the same drip irrigation fertilization level. There was no significantly variance in the content of GAs and ETH between BMM and SRM under different drip irrigation levels. However, GA and ETH under BML treatment were significantly lower than those under SRL treatment.

### 3.5 Dry matter and yield components

The dry matter before silking stage showed significant differences among various mulching materials using drip irrigation fertilization irrigation strategies (**Table 5**), and under BMH and SRH treatments, compared to all other treatments using drip irrigation fertilization strategies, the dry matter after silking stage was significantly highest (**Table 6**). Under BMH treatment, compared with other treatments, the total dry matter before and after silking stage was significantly increased. The mean of two year data shows that the total dry matter of plants treated with BMH and SRH significantly improved by 44.7% and 43.3% compared to SRL. The drip irrigation fertilization strategy significantly affects maize ear traits. With the improvement of drip irrigation fertilization level, the ear length, ear diameter, ear grain number, ear grain weight, and thousand-grain weight of maize revealed a significantly increase trend under different film covering materials. Pearson’s correlation coefficients of the endogenous hormonal changing, evapotranspiration, grain yield, soil water storage, soil respiration rate and water use efficiency of maize showed in **Table 7**. Significant positive correlations were observed between GY, IAA, SR, SWS, TGW, WUE, and Z+ZR contents. Moreover, significant negative correlations were observed between ETH, ET, GAs, ABA with GY, IAA, SR SWS, TGW, WUE and Z+ZR contents. Due to the highest yield composition and water use efficiency, biodegradable film coverage on ridges with higher drip irrigation levels can yield the highest grain yield. The highest yield (54.0%) and water use efficiency (60.7%) of BMH treatment were higher than those of SRL treatment due to its higher yield composition and efficient drip irrigation water use.

**Table 5:** Effects of different treatments on ear characteristics of maize during 2023 and 2024.

Treatments	Ear length (cm)	Ear diameter (mm)	No. of grains (ear^-1^)	Grain weight (ear^-1^)	1000-grain weight (g)
2023					
BMH	17.5a	50.2a	548.4a	144.2a	327.5a
BMM	16.0a	49.1a	484.6c	130.6c	295.6b
BML	11.8c	48.5b	317.9e	93.0e	286.9c
SRH	16.8a	49.6a	516.5b	137.4b	323.1a
SRM	15.0b	47.1b	451.0d	121.4d	277.1d
SRL	10.1c	46.7c	239.0f	83.1f	251.0e
ANOVA					
CM	*	*	*	*	*
DI	*	*	*	*	*
CM * DI	ns	ns	ns	ns	ns
2024					
BMH	18.8a	51.0a	558.3a	147.1a	289.9a
BMM	18.2a	49.8a	524.5b	141.9b	265.8c
BML	12.8c	47.7b	342.1e	95.6e	250.9d
SRH	17.7a	49.8a	474.4c	132.9c	279.9b
SRM	15.8b	48.4b	465.2d	128.3d	246.6d
SRL	11.0c	47.3b	285.0f	79.9f	227.0e
ANOVA					
CM	*	*	*	*	*
DI	*	**	*	*	*
CM * DI	ns	ns	ns	ns	ns

Followed by different small letters within a column indicate significantly different at the 5% probability level (LSD; n=3). ** Significant differences at p< 0.01; * significant differences at p< 0.05; ns indicates non-significant difference.

**Table 6:** Effects of different treatments on dry matter (g plant^-1^), evapotranspiration rate, grain yield and water use efficiency of maize during 2023 and 2024.

Treatments	Total DM (g plant^-1^)	Pre-silking DM (g plant^-1^)	Post-silking DM (g plant^-1^)	ET (mm)	Grain yield (t ha^-1^)	WUE (Kg ha^-1^ mm^-1^)
2023						
BMH	381.5a	160.4a	222.0a	354.9e	12.5a	35.1a
BMM	345.4c	147.4b	198.8b	388.7c	10.3b	26.5c
BML	268.5e	120.0d	149.3d	432.6a	7.9d	18.3d
SRH	371.5b	151.6b	220.6a	375.5d	11.7b	31.1b
SRM	326.5d	144.5c	182.8c	379.2c	9.6c	25.3c
SRL	225.2f	110.2e	115.9e	418.5b	6.3e	15.1e
ANOVA						
CM	*	*	*	*	**	**
DI	*	*	*	*	**	**
CM * DI	ns	ns	ns	ns	ns	ns
2024						
BMH	356.4a	148.4a	208.8a	324.3d	12.8a	39.4a
BMM	284.1c	126.4c	158.5b	339.4c	10.9c	32.1c
BML	211.5e	97.5d	114.8d	352.1b	7.5e	21.3d
SRH	348.4b	145.9a	203.4e	337.2c	11.9b	35.3b
SRM	276.5d	123.9c	153.4c	358.1b	8.0d	22.4d
SRL	182.7f	89.6e	93.8e	372.3a	5.3f	14.2e
ANOVA						
CM	*	*	*	*	**	**
DI	*	**	*	*	**	**
CM * DI	ns	ns	ns	ns	ns	ns

Followed by different small letters within a column indicate significantly different at the 5% probability level (LSD; n=3). ** Significant differences at p< 0.01; * significant differences at p< 0.05; ns indicates non-significant difference.

**Table 7:** Pearson’s correlation coefficients among different parameters of maize.

	ABA	ET	ETH	GAs	GY	IAA	SR	SWS	TGW	WUE
ET	-0.387									
ETH	-0.954**	0.409**								
GAs	-0.967**	0.421	0.947**							
GY	0.943**	-0.553*	-0.951**	-0.946**						
IAA	0.950**	-0.385	-0.928**	-0.980**	0.935**					
SR	0.946**	-0.194	-0.936**	-0.946**	0.888**	0.946**				
SWS	0.837**	-0.554*	-0.900**	-0.858**	0.904**	0.886**	0.805**			
TGW	0.863**	-0.632*	-0.858**	-0.903**	0.929**	0.913**	0.791**	0.917**		
WUE	0.886**	-0.715*	-0.895**	-0.881**	0.975**	0.863**	0.781**	0.889**	0.925**	
Z+ZR	0.993**	-0.361	-0.952**	-0.969**	0.945**	0.953**	0.942**	0.848**	0.856**	0.882**

ABA, abscisic acid; ET, evapotranspiration; ETH, ethylene; GAs, gibberellins; GY, grain yield; IAA, indole-3-acetic acid; SR, soil respiration rate; SWS, soil water storage; TGW, thousand grains weight; WUE, water use efficiency; Z+ZR, zeatin+zeatin riboside.

**Significant at the 0.01 probability level.

*Significant at the 0.05 probability level.

## 4 Discussion

Drought is a vital factor limiting maize productivity in rainwater irrigation areas (Liu et al., 2021), while rainfall mainly affects the main source of water in dry farming systems (Ma et al., 2012). The water use efficiency of crops increases with crop development, but no drought conditions were recorded under BMH and BMM treatments. Under BMH treatment at a soil depth of 0-200cm, the SWS of different growth stages (excluding the SS stage) were significantly greater as compared with all other treatments. Compared with flat cultivation, biodegradable film coverage reduces evaporation and can effectively save more soil precipitation (Zhang et al., 2011; Memon et al., 2021). We found that the SWS trend of each treatment significantly increased from the SS phase to the MS phase. With the improvement of drip irrigation fertilization levels under different film-covering materials, SWS has increased throughout the entire growth stage of maize. Previous studies suggested the use of RF planting systems as this approach significantly increases soil water storage by withholding rainwater from mild rainfall events and decreasing ET concentrations, which improves agricultural productivity (Gao et al., 2000). Under the use of biodegradable film-covered ridges and drip irrigation strategies resulted in the lowest ET rate compared to those under soil crust ridges. ET significantly decreased with the increase of BMH; BMM; BML; SRH and SRM treatment. These studies also indicate that these conditions raise SWS, decrease soil evaporation, and significantly increase maize production (Wu et al., 2008; Li and Gong, 2002). During the wet growing season, rain storms reduce soil breathing rate by 29%, indicating that soil water content and temperature together affected soil breathing rate (Ali et al., 2017). Chen et al. (2011) also study that the soil breathing rate increased significantly as the lack of irrigation increased, but excessive irrigation significantly reduced the soil breathing rate. There was no significant difference in soil respiration rate between BMH and BMM treatments in the FS, GFS, and MS phases.

The effective filling time of the seeds determines the final harvest. This grain filling process is not only genetically calculated but also influenced by ecological factors such as sowing time, nutrient level, cultivation, and mulching method (Fang et al., 2006; Qiang et al., 2022). Early studies have shown that, compared to flat cultivation without plastic film cover, the RF system with nutrition strategies promotes the grain filling in advance, improving the maize seed filling process and final grain weight (Ali et al., 2017). In our findings that under various films covering materials, W_max_, G_mean_ and G_max_ increased with the increase of drip fertilization level (**Table 3**). During 2023, there was no significantly variance in W_max_, G_mean_ and G_max_ between BMH and BMM treatments under different drip irrigation levels. However, under different film mulching materials, W_max_, G_mean_ and G_max_ under low drip irrigation levels were significantly lower as compared with high and medium drip irrigation levels. An early study showed that under dryland farming systems, the weight of maize seeds was significantly affected (Zhang et al., 2009; Thompson, 2023). The maximum filling occurrence time (T_max_) of BMH and BMM treatments was significantly later than other treatments. AGP increases with the improvement of drip irrigation levels under different film-covering materials. Under different mulching materials, the AGP under high drip irrigation fertilization level was significantly higher than that under low drip irrigation fertilization level. Ali et al. (2017) believe that compared to traditional flooded cultivation, non-flooded plastic film significantly reduces the seed filling and grain weight of low-quality rice. The seed filling process significantly improved in the early stage of silking and then decreased. Under BMH and BMM treatments, the seed-filling process was significantly higher. The seed filling process of BM and SR treatments with high and medium drip irrigation fertilization levels was significantly increased. The BM treatment with high or moderate drip irrigation fertilization levels showed the highest grain filling rate 21 days after silking. An early study showed that the RF system can increase the seed filling process, thereby increasing soil moisture content, indicating that seed filling processes are extremely sensitive to water stress (Xu et al., 2007).

Grain filling process is significantly regulated by cytokines (CTK), and it has been reported that CTK in maize ears correlates significantly with cereal development (Zhang et al., 2009, 2022). CTK is usually the highest in maize endosperm seeds, and may be necessary for early cell division during growth (Ali et al., 2016). The content of CTK and IAA also plays an important role in regulating the seed-filling process (Yang et al., 2002; He et al., 2022). The Z+ZR and IAA concentrations of maize vary similarly during the filling process. The Z+ZR and IAA content of maize increased briefly during the filling phase, reached its maximum 18 days after silking, and gradually decreased thereafter. In the early stages of seed filling, Z+ZR and IAA in seeds grow rapidly and then decrease. In BMH, Z+ZR and IAA improved significantly compared to other treatments. BM with high drip irrigation also significantly improved Z+ZR and IAA in the initial phase of the patch. However, compared to SR implantation technique, peak Z+ZR and IAA values in BM implantation mode are synchronous. Ali et al. (2017) study that in the early stages of silk Z+ZR and IAA can normalize maize seed filling, most likely by controlling endosperm cell distribution, resulting in sinking intensity.

Ali et al. (2017) study that ABA increased ETH to control the seed filling process. In pea plants, rapid elongation of the pods is due to the fact that the endosperm has the highest concentration of GA_S_ (Ali et al., 2016; Huang et al., 2021). The relatively high panicles of rice before and during flowering are due to the high GA_S_ content (Eeuwens and Schwabe, 1975; Katimbo et al., 2022). The ABA of maize grains increases rapidly during the filling phase and gradually decreases. As compared with SRL and BML treatments, the ABA content of BMH treatment is significantly higher. Compared with SRL and BML treatments, BM treatment significantly increased ABA content at high and moderate drip irrigation fertilization levels. However, under different film drop fertilization strategies, the effect of different film covering materials on ABA was not significant from 33 to 42 days after silking stage. Kurogochi et al. (1979) study that ABA promotes and ETH inhibit the seed filling process. The trends of GA and ETH are similar during the patch season. Gaseous and ETH compounds in maize are gradually reduced throughout the filling process. During the whole seed filling process, the GAs and ETH values of SRL and BML were significantly greater as compared with BMH and SRH. GA also participates in regulating the grain filling and development. Compared to BM treatment of low drip irrigation strategy SR treatment of low drip irrigation strategy significantly improved the evolution of GA and ETH. However, BML-treated GA and ETH were significantly lower than SRL-treated patients. This finding is similar to a previous study (Martinez et al., 2011; Xu et al., 2020).

Earlier research work have shown that RF systems can improve SWS, temperature, and nutrients uptake and are an effective way to improve yields and WUE (Yang et al., 2006; Ren et al., 2008). The yield of maize is linked to the hormonal changes in maize. The total dry matter of plants treated with BMH and SRH treatment increased significantly by 44.7% and 43.3% compared to SRL treatment. With the improvement in drip irrigation, the yield composition ratio of maize under different film coating materials shows a significantly increasing trend. Mulching materials can increase the availability of water to plants more than flat planting; to maximize WUE and food production (Blanco-Moure et al., 2012; Zhang et al., 2021). Due to the highest yield composition and water use efficiency, biodegradable film coverage on ridges with higher drip irrigation levels can yield the highest grain yield. In addition, we found that drip irrigation significantly improved yield and reduced ET rate, thus efficiently enhancing the balance of hormone changes.

## 5 Conclusion

The current research results clearly indicate that compared with the SR treatment under a mulch-based drip fertigation strategy, BMH treatment significantly improved the grain filling rate, endogenous hormone levels, dry matter accumulation, WUE, yield, and yield components of maize. Grain filling has a significant impact on grain yield. The BMH treatment significantly increased soil water storage, as well as the concentrations of endogenous hormones, including ABA, Z+ZR, and IAA, while decreasing the levels of GAs and ETH, thereby increasing maize yield. These results indicate that BMH treatment improved the grain filling process, soil respiration rate, WUE, yield, and yield components of maize. High drip fertigation combined with BM or SR treatment significantly altered endogenous hormone levels, thereby regulating the AGP, G_max_, W_max_, G_mean_ and T_max_ in maize. In addition, during the grain filling period, both high-rate drip fertigation and the BM strategy significantly increased soil water storage and soil respiration rate. These findings indicate that BMH treatment represents a promising strategy for dryland agricultural systems because it significantly enhances soil water storage, WUE, and total dry matter accumulation, thereby regulating maize grain filling. This process was significantly correlated with the balance of endogenous hormones in the grains, defined as the quantitative relationship among growth-promoting hormones (IAA and GA_3_) and stress-related hormones ABA, which collectively regulate grain development, plant growth and adaptation to environmental stress. Future studies are needed to investigate the impact of ridge-furrow (RF) systems on crop production, runoff use efficiency and economic returns under different ridge–furrow ratios, ridge-covering materials, soil types, slopes and plant species using biodegradable mulching materials such as grass, hay, wood chips, wood fibers and straw.

## Data Availability

The original contributions presented in the study are included in the article/supplementary material. Further inquiries can be directed to the corresponding author.
